# Age-Dependent Progression of SARS-CoV-2 Infection in Syrian Hamsters

**DOI:** 10.3390/v12070779

**Published:** 2020-07-20

**Authors:** Nikolaus Osterrieder, Luca D. Bertzbach, Kristina Dietert, Azza Abdelgawad, Daria Vladimirova, Dusan Kunec, Donata Hoffmann, Martin Beer, Achim D. Gruber, Jakob Trimpert

**Affiliations:** 1 Institut für Virologie, Freie Universität Berlin, Robert-von-Ostertag-Str. 7-13, 14163 Berlin, Germany; no.34@fu-berlin.de (N.O.); luca.bertzbach@fu-berlin.de (L.D.B.); azza.abdelgawad@fu-berlin.de (A.A.); daria.vladimirova@fu-berlin.de (D.V.); dusan.kunec@fu-berlin.de (D.K.); 2 Institut für Veterinärpathologie, Freie Universität Berlin, Robert-von-Ostertag-Str. 15, 14163 Berlin, Germany; kristina.dietert@fu-berlin.de (K.D.); achim.gruber@fu-berlin.de (A.D.G.); 3 Tiermedizinisches Zentrum für Resistenzforschung, Freie Universität Berlin, 14195 Berlin, Germany; 4 Institut für Virusdiagnostik, Friedrich-Loeffler-Institut, Südufer 10, 17493 Greifswald-Insel Riems, Germany; donata.hoffmann@fli.de (D.H.); martin.beer@fli.de (M.B.)

**Keywords:** coronavirus, *Mesocricetus auratus*, animal model, COVID-19, pneumonia, age-related disease, histopathology, in situ hybridization, cellular tropism, serology

## Abstract

In late 2019, an outbreak of a severe respiratory disease caused by an emerging coronavirus, SARS-CoV-2, resulted in high morbidity and mortality in infected humans. Complete understanding of COVID-19, the multi-faceted disease caused by SARS-CoV-2, requires suitable small animal models, as does the development and evaluation of vaccines and antivirals. Since age-dependent differences of COVID-19 were identified in humans, we compared the course of SARS-CoV-2 infection in young and aged Syrian hamsters. We show that virus replication in the upper and lower respiratory tract was independent of the age of the animals. However, older hamsters exhibited more pronounced and consistent weight loss. In situ hybridization in the lungs identified viral RNA in bronchial epithelium, alveolar epithelial cells type I and II, and macrophages. Histopathology revealed clear age-dependent differences, with young hamsters launching earlier and stronger immune cell influx than aged hamsters. The latter developed conspicuous alveolar and perivascular edema, indicating vascular leakage. In contrast, we observed rapid lung recovery at day 14 after infection only in young hamsters. We propose that comparative assessment in young versus aged hamsters of SARS-CoV-2 vaccines and treatments may yield valuable information, as this small-animal model appears to mirror age-dependent differences in human patients.

## 1. Introduction

Emerging coronaviruses have caused serious global public health concerns in the past two decades and cause infections that lead to severe respiratory and occasionally systemic disease [1]. These include severe acute respiratory syndrome (SARS)-CoV as well as Middle East respiratory syndrome (MERS)-CoV, both of which resulted in high morbidity and mortality in infected humans [2,3]. Similar to other emerging CoV, the novel SARS-CoV-2 likely arose from an ancestor in bats and amplified in a yet unknown animal reservoir before making its jump into the human population [4].

SARS-CoV-2 has pushed global health systems to the brink of breakdown. The remarkably fast and unexpected spread of SARS-CoV-2 can be attributed to efficient replication in the upper respiratory tract and robust human-to-human transmission. While COVID-19 is primarily a respiratory syndrome, it can induce quite variable clinical signs including fatigue, headache, and gastrointestinal symptoms, which can in severe cases result in fatality [5]. It has become evident that differences in the type and severity of SARS-CoV-2-induced disease and its sequelae seem to be strongly correlated with the age of patients and exacerbated by pre-existing medical conditions (e.g., chronic obstructive pulmonary disease, heart disease, diabetes, obesity) [1,6,7,8].

The availability of reliable animal models is of critical importance for pathogenesis studies as well as the development and preclinical evaluation of vaccines and therapeutics [2,9,10]. For SARS-CoV-2, the susceptibility of several animal species was predicted by in silico analysis based on comparisons of the entry receptor for SARS-CoV and SARS-CoV-2, angiotensin converting enzyme 2 (ACE2). These predictions are of relevance because the ACE2 sequence is deemed an important factor governing susceptibility [11]. More specifically, the interaction of the viral spike (S) glycoprotein receptor binding domain with its ACE2 counterpart was examined [12,13], and in some cases confirmed in vivo [9]. Productive SARS-CoV-2 infection was shown in non-human primates, which developed respiratory disease recapitulating moderate disease as observed in humans [14,15,16,17]. Mice are not naturally susceptible to SARS-CoV-2, but mouse-adapted virus strains have been developed and used in BALB/c mice [18,19]. Moreover, transgenic mice expressing human ACE2 represent a lethal SARS-CoV-2 infection model resulting in significant weight loss and permitting robust virus replication in the respiratory tract including the lungs [20]. Ferrets have provided valuable data in the case of SARS-CoV [21,22], and two studies describe the infection of ferrets with SARS-CoV-2 and successful transmission to in-contact animals without clinical signs [23,24]. 

First and preliminary studies also focused on the assessment of a Syrian hamster model that had previously been used successfully in SARS and MERS research [21,22,25,26]. It was suggested that hamsters are highly susceptible, although they were reported to show no or only moderate respiratory signs and body weight losses. However, it is important to note that only young male hamsters of 4 to 5 weeks of age were used in these studies [27,28]. 

We sought to explore age-related differences in the course of SARS-CoV-2 infection in Syrian hamsters and to establish a small-animal model that resembles the more severe SARS-CoV-2-infection observed particularly in elderly patients.

## 2. Materials and Methods

### 2.1. Ethics Statement

We conducted all animal work in compliance with relevant national and international guidelines for care and humane use of animals. The animal use protocol for the experiments reported here was approved by the Landesamt für Gesundheit und Soziales in Berlin, Germany (approval number 0086/20; approved on 30.04.2020).

### 2.2. Viruses and Cells

Virus stocks were prepared from a previously published SARS-CoV-2 isolate (BetaCoV/Germany/BavPat1/2020) [29], which was kindly provided by Drs. Daniela Niemeyer und Christian Drosten, Charité Berlin, Germany. The isolate, referred to as SARS-CoV-2 München (SARS-CoV-2M) [30], was handled under the appropriate safety precautions in a BSL-3 facility (Freie Universität Berlin, Institut für Virologie) and propagated on Vero E6 cells (ATCC CRL-1586) in minimal essential medium (MEM; PAN Biotech, Aidenbach, Germany) supplemented with 10% fetal bovine serum (PAN Biotech), 100 IU/mL penicillin G and 100 µg/mL streptomycin (Carl Roth, Karlsruhe, Germany).

### 2.3. Animal Husbandry

Thirty-six 6- or 32-to-34-week-old female and male Syrian hamsters (Mesocricetus auratus; breed RjHan:AURA, Janvier Labs, Saint-Berthevin, France) were kept in individually ventilated cages (IVCs; Tecniplast, Buguggiate, Italy) in an approved BSL-3 facility. IVCs were equipped with enrichment (Carfil, Oud-Turnhout, Belgium). All animals had unrestricted access to food and water and were allowed to acclimate to the conditions for seven days prior to infection. Cage temperatures and relative humidities were recorded daily and ranged from 22–24 °C and 40–55%, respectively.

### 2.4. Animal Experiments 

The 6-week-old hamsters were randomly distributed into two groups: mock (*n* = 12, 6-week-old) and young infected (*n* = 12, 6-week-old). The third group represents the aged infected hamsters (*n* = 12, 32–34-week-old). IPTT-300 transponders (BioMedic Data Systems, Seaford, DE, USA) were subcutaneously implanted into all hamsters 2 days prior to infection to allow the identification and monitoring of body temperatures. Animals were mock-infected with 60 µL medium from uninfected Vero E6 cells or infected with 1 × 105 pfu SARS-CoV-2M in 60 µL by intranasal instillation. For transponder implantation, hamsters were sedated with butorphanol (2.5 mg/kg; CP-Pharma, Burgdorf, Germany) and midazolam (2 mg/kg; Braun, Melsungen, Germany). For infections, hamsters were sedated with ketamine (25 mg/kg; Serumwerk Bernburg, Bernburg, Germany) and midazolam (2 mg/kg; Braun).

On 2, 3, and 5 days post-infection (dpi), three randomly assigned hamsters of each group were euthanized by exsanguination under medetomidine (0.15 mg/kg; Pharma-Partner, Hamburg, Germany), midazolam (2 mg/kg), and butorphanol (2.5 mg/kg) anesthesia [31]. Blood, nasal washes, bucco-laryngeal swabs, lungs (left and right), kidneys, spleens, duodenums, and blood sera were collected for (histo)pathological examinations and/or virus titrations, RT-qPCR, and serological examination. During the 14-day experiment, body temperatures, body weights, and clinical signs of all animals were monitored twice daily. Animals that had a body weight loss of more than 10% weight over a 72 h period were euthanized in compliance with the animal use protocol. Such humane termination applies to the two hamsters euthanized 7 dpi.

### 2.5. Histopathological Examination

For histopathology and in situ hybridization (ISH), the left lung lobe was carefully removed, immersion-fixed in formalin, pH 7.0, for 48 h, embedded in paraffin, and cut in 2 μm sections. For histopathology, slides were stained with hematoxylin and eosin (HE) after dewaxing in xylene and rehydration in decreasing ethanol concentrations. Lung sections were microscopically evaluated in a blinded fashion by a board-certified veterinary pathologist to assess the character and severity of pathologic lesions using lung-specific inflammation scoring parameters as described for other lung infection models before [32]. Three different scores were used that included the following parameters: (1) lung inflammation score including severity of (i) interstitial pneumonia (ii) bronchitis, (iii) epithelial necrosis of bronchi and alveoli, and (iv) hyperplasia of type II-alveolar epithelial cells; (2) immune cell infiltration score taking into account the presence of (i) neutrophils, (ii) macrophages, and (iii) lymphocytes in the lungs as well as (iv) perivascular lymphocytic cuffing; and (3) edema score including (i) alveolar edema and (ii) perivascular edema. 

ISH was performed as reported previously [33] using the ViewRNA™ ISH Tissue Assay Kit (Invitrogen by Thermo Fisher Scientific, Darmstadt, Germany) following the manufacturer’s instructions with minor adjustments. Probes for the detection of N gene RNA of SARS-CoV-2 (NCBI database NC_045512.2, nucleotides 28,274 to 9533, assay ID: VPNKRHM) and the mouse housekeeping gene eukaryotic translation elongation factor-1α (EF1a; assay ID: VB1-14428-VT, Affymetrix, Inc., Santa Clara, CA, USA), which shares 95% sequence identity with the Syrian hamster orthologue, were designed. Lung sections (2 µm thickness) on adhesive glass slides were dewaxed in xylol and dehydrated in ethanol. Tissues were incubated at 95 °C for 10 min with subsequent protease digestion for 20 min. Sections were fixed with 4% paraformaldehyde in phosphate-buffered saline (Alfa Aesar, Thermo Fisher, Kandel, Germany) and hybridized with the probes. Amplifier and label probe hybridizations were performed according to the manufacturer’s instructions using fast red as the chromogen, followed by counterstaining with hematoxylin for 45 s, washing in tap water for 5 min, and mounting with Roti^®^-Mount Fluor-Care DAPI (4, 6-diaminidino-2-phenylindole; Carl Roth). For negative and morphologically intact controls, lungs from uninfected hamsters of each group (*n* = 4) were included. In addition, an irrelevant probe for the detection of pneumolysin was used as a negative control for unspecific reactions. HE-stained and ISH slides were analyzed and images were taken using an Olympus BX41 microscope with a DP80 Microscope Digital Camera and the cellSens™ Imaging Software, version 1.18 (Olympus Corporation, Münster, Germany). For the display of overviews of whole lung lobe sections, slides were automatically digitized using the Aperio CS2 slide scanner (Leica Biosystems Imaging Inc., Vista, CA, USA), and image files were generated using the Image Scope Software (Leica Biosystems Imaging Inc.).

The percentages of lung tissues affected by inflammation were determined histologically by an experienced board certified experimental veterinary pathologist (K.D.) as described previously [34]. Lung inflammation scores were determined as (0) absent, (1) minimal, (2) mild, (3) moderate, or (4) severe, and quantified as described previously [35]. Immune cell influx scores and edema scores were rated from (0) absent to (1) sporadic, (2) mild, (3) moderate, or (4) severe.

ISH signals were digitally quantified on scanned whole slides of each animal using the Aperio positive pixel count algorithm (Leica Biosystems Imaging Inc.) with minor adjustments. Specifically, a positivity score encompassing the number of positive pixels in relation to the total number of pixels per tissue scan as well as the total intensity of positive pixels were determined (Figure S3).

### 2.6. Virus Titrations

For an assessment of virus titers from 25 mg of lung tissue, tissue homogenates were serially diluted and plated on Vero E6 cells in 12-well cell culture plates (Sarstedt, Nümbrecht, Germany). At 3 dpi, cells were fixed in 4% formalin, stained with 0.1% crystal violet (in 25% methanol), and plaques were counted. 

### 2.7. RNA Extractions and Quantitative RT-PCR

RNA was extracted from nasal washes and tracheal swabs with the RTP DNA/RNA Virus Mini Kit (Stratec, Birkenfeld, Germany) according to the manufacturer’s instructions. The innuPREP Virus DNA/RNA Kit (Analytic Jena, Jena, Germany) was used for RNA extractions from tissue samples. Viral RNA was quantified using a one-step RT qPCR reaction with the NEB Luna Universal Probe One-Step RT-qPCR (New England Biolabs, Ipswitch, MA, USA) and the 2019-nCoV RT-qPCR primers and probe (E_Sarbeco) [36] on a StepOnePlus RealTime PCR System (Thermo Fisher Scientific, Waltham, MA, USA) according to the manufacturer’s instructions. Viral RNA copies were then normalized to cellular RPL18 as previously described [37]. All primers and probes are listed in Table S2.

Standard curves for absolute quantification were generated from serial dilutions of SARS-CoV-2 RNA obtained from a full-length virus genome cloned as a bacterial artificial chromosome and propagated in E. coli or from serial dilutions of the purified hamster RPL-18 PCR product. The latter was generated by PCR using RPL-18 qPCR-primers (Table S2) and a cDNA template obtained from hamster lung tissue. In lung tissue, viral RNA copies were calculated per 1×10^5^ hamster RPL-18 transcripts.

### 2.8. Serology

For serum neutralization assays, 50 µL of medium containing 103.3 tissue culture infectious doses 50 (TCID_50_) of SARS-CoV-2M were mixed with 50 µL of diluted serum. Each sample was tested in triplicate. After 1 h incubation at 37 °C, the mixture was transferred to confluent Vero E6 cells in a 96-well plate (Sarstedt, Nümbrecht, Germany). Viral replication was assessed after 3 days by the detection of cytopathic effects.

### 2.9. Statistical Analyses

Statistical analyses were performed using Graph-Pad Prism v8 (GraphPad Software Inc., San Diego, CA, USA). The statistical details of all analyzed experiments can be found in the respective figure legend.

## 3. Results and Discussion

**Figure 1:**
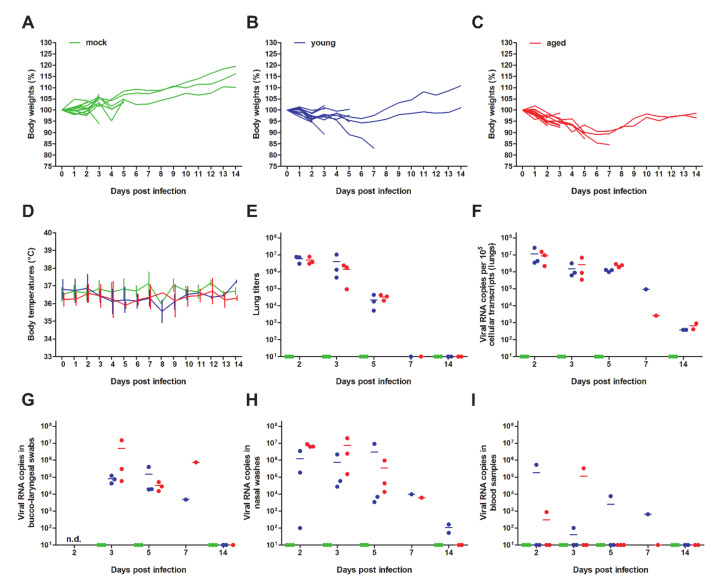
Body weight changes, body temperatures, and viral loads of young versus aged Syrian hamsters infected intranasally with severe acute respiratory syndrome (SARS)-CoV-2. Individual relative body weights of (**A**) mock-infected, (**B**) young, and (**C**) aged hamsters over the course of 14 days after infection are given. Kruskal–Wallis tests with Dunn’s multiple comparison post hoc tests for each time point revealed significant differences at 2 dpi (mock vs. young; *p* < 0.01) and 2–5 dpi (mock vs. old; *p* < 0.01). (**D**) Temperature changes (as means with SD). Viral loads were determined from homogenized right cranial lung lobes. (**E**) Virus titers of 25 mg of lung tissue determined by plaque assay in Vero E6 cells, and (**F**) corresponding virus genome copy numbers as determined by RT-qPCR. Viral loads were also determined by RT-qPCR in (**G**) bucco-laryngeal swabs, (**H**) nasal washes and (**I**) 25 µL of whole blood samples. The color codes represent mock-infected (green), infected young (6-week-old, blue) and aged hamsters (33- to 35-week-old, red).

**Figure 2:**
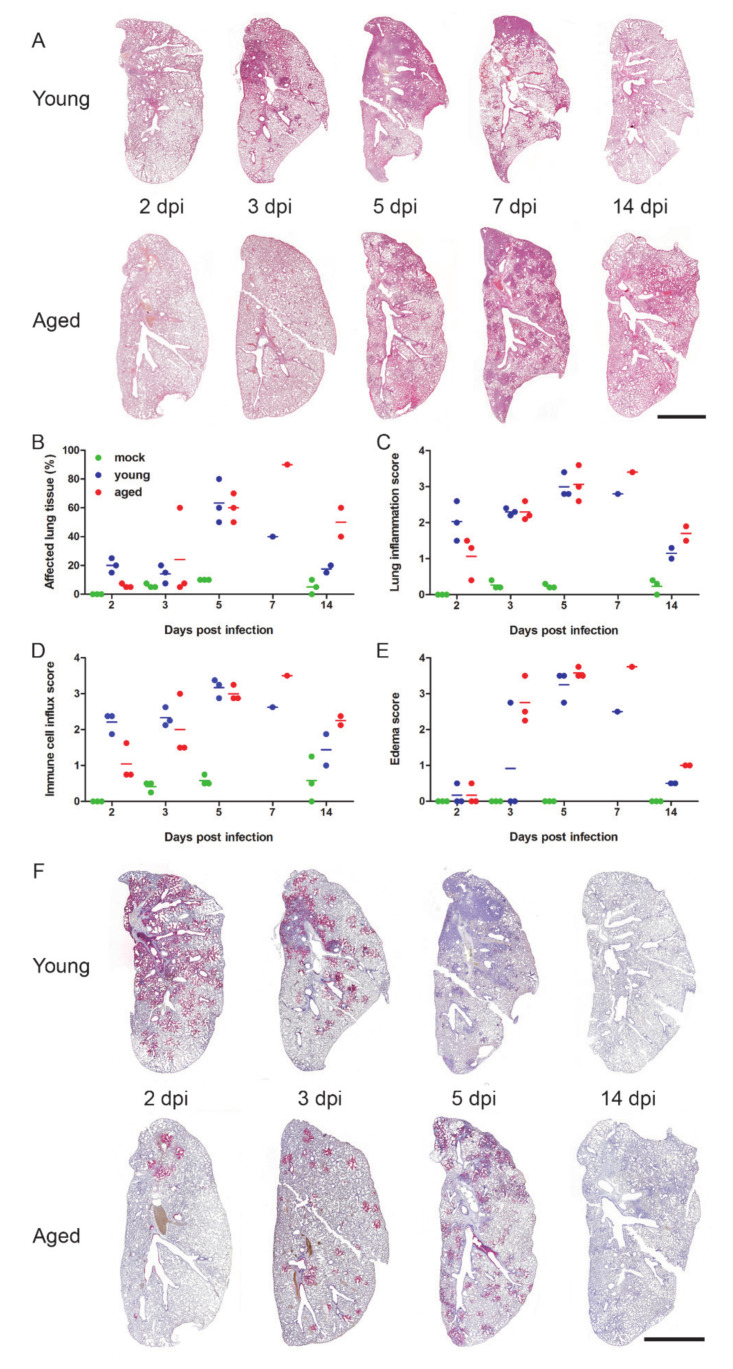
Lung histopathology (**A–E**) and detection of SARS-CoV-2 RNA (**F**) at different time points after infection. (**A**) Time-dependent course of pneumonia in young and adult hamsters representative of each group (*n* = 3 for 2, 3, and 5 dpi, *n* = 1 for 7 dpi and *n* = 2 for 14 dpi; Bar = 0.5 cm) (**B**) Affected areas of inflammation were identified histologically for each lung and compared between the groups. (**C**) Lung inflammation score taking into account (i) severity of pulmonary inflammation; (ii) bronchitis (iii) bronchial and alveolar necrosis; iv) hyperplasia of alveolar epithelial cells type II. (**D**) Immune cell influx score taking into account the infiltration of lung tissue with (i) neutrophils; (ii) macrophages; (iii) lymphocytes; (iv) perivascular lymphocytic cuffing; and (**E**) edema score including (i) alveolar and (ii) perivascular edema. Scores and parameters in C to E were graded as absent (0), minimal (1), mild (2), moderate (3), or severe (4) as described [35]. (**F**) Time-dependent distribution of SARS-CoV-2 RNA signals in young and adult hamsters representative of each group as detected by in situ hybridization (group sizes as in A; for digital image analysis of the differences, see Figure S3; bar = 0.5 cm).

For our experiments, we used a total of 36 female and male Syrian hamsters (*Mesocricetus auratus*), which were either 6 (*n* = 24) or 32 to 34 weeks old (*n* = 12). Hamsters were kept in individually ventilated cages (IVCs) and randomly assigned to three groups: mock (*n* = 12, 6-week-old), young infected (*n* = 12, 6-week-old) and aged infected (*n* = 12, 32- to 34-week-old). Animals were mock-infected with supernatants of cell culture medium taken from uninfected Vero E6 cells or infected with 1 × 10^5^ plaque-forming units of SARS-CoV-2 München (SARS-CoV-2M; BetaCoV/Germany/ BavPat1/2020) [30]. During the 14-day experiment, body temperatures, body weights, and clinical signs were recorded daily. Animals were euthanized and sampled at different time points after infection to assess virus titers in various organs and to examine pathological changes in the lungs (Figure 1 and Figure 2). 

First, we observed age-dependent SARS-CoV-2-induced body weight losses, with more pronounced weight reductions in aged compared to young hamsters (Figure 1A–C). Mean body weight losses peaked at 6 to 7 days post-infection (dpi), with partial recovery until 14 dpi in both infected groups. There were no differences in body temperatures between the infected groups or between infected and mock-infected animals (Figure 1D).

Next, we determined viral titers and SARS-CoV-2 RNA copy numbers in various tissues by RT-qPCR [36,37] and performed virus titrations from lung homogenates using Vero E6 cells (Figure 1E–H). Results were similar between age groups and confirmed high viral loads in respiratory samples at early time points after infection, but a relatively rapid clearance of infection. While we did not identify any other sex-specific differences, it seems important to note that the RT-qPCR of blood samples revealed viremia in two male individuals from both age groups with viral RNA copy numbers of >10^5^ at 5 and 7 dpi, respectively (Figure 1I). In these individuals, we also detected relatively high levels of viral RNA in the spleen, kidneys, and duodenum, indicating a systemic spread of SARS-CoV-2 in some cases (Table S1). To further investigate the potential dissemination of infection, we tested the aforementioned organs from all animals sacrificed at 5 dpi and found them to be either negative for SARS-CoV-2 RNA or to contain only low levels of viral RNA (Table S1).

Viral loads in bucco-laryngeal swabs and nasal washes appeared to be a reliable surrogate of the presence or absence of virus in the lungs in both age groups. The average loads ranged between 10^4^ and 10^7^ copies, respectively, at early times after infection, indicating that these sampling techniques can be used to monitor SARS-CoV-2 replication in Syrian hamsters (Figure 1G,H).

At 14 dpi, hamsters had mounted a humoral immune response as evidenced by relatively high titers of neutralizing antibodies. It is worth noting that antibody titers appear to be higher in young when compared to aged hamsters in the animals tested (Table S1). 

Histopathology revealed clear age-dependent differences, with young hamsters launching an earlier and stronger immune cell influx into the lungs associated with a faster recovery than their aged counterparts (Figure 2A–E). At 2 dpi, young hamsters developed a marked necro-suppurative bronchointerstitial pneumonia with strong alveolar and interstitial influx of neutrophils and macrophages as well as perivascular lymphocytic cuffing, which was much milder or absent in the aged group (Figure S1A, right panel). In contrast, only aged animals developed pronounced alveolar and perivascular edema indicating vascular leakage at 3 dpi (Figure 2E and Figure S2C). A more diffuse, severe bronchointerstitial pneumonia was similarly present in both groups at 5 dpi with an onset of tissue regeneration, including hyperplasia of the bronchial epithelium (Figure S1C, arrowhead) and type II alveolar epithelial cells. 

Only at the 5 dpi time point was the arterial and venous endothelium of animals in both groups swollen and vacuolated, with necrotic endothelial cells separated from the underlying basement membrane by the presence of subendothelial lymphocytes and neutrophils (Figure S2C, left), which was consistent with what has been described as endothelialitis in human SARS-CoV-2 infection [38]. At 7 dpi, recovery as indicated by the marked hyperplasia of bronchial epithelial cells and type II alveolar epithelial cells were seen in both groups (Figure S2A). Interestingly, lung tissues had almost recovered in young hamsters at day 14, while the aged animals still had persistent tissue damage and active inflammation (Figure S2B).

From 2 dpi onwards, SARS-CoV-2 RNA was detected by in situ hybridization in bronchial epithelial cells, debris in the bronchial lumen, alveolar epithelial cells type I and type II, as well as macrophages in both groups, again with clear age-dependent differences over time (Figure 2F, Figures S2D and S3). Young animals had high amounts of viral RNA in numerous bronchial epithelial cells and within the bronchial lumen that was accompanied by marked spreading through the lung parenchyma on 2 and 3 dpi. In contrast, aged animals had less virus RNA present in the bronchi. We detected only a scattered pattern of infected bronchial epithelial cells and sporadic areas of parenchymal infection at 2 and 3 dpi. At 5 dpi, viral RNA was undetectable in the bronchi of young hamsters, and only small infected areas containing low levels of RNA with a patchy distribution were detected. It is noteworthy that aged animals, at the same time after infection, had increased numbers of infected areas with a similarly patchy distribution throughout the lungs as well as copious amounts of viral RNA associated with cellular debris in the bronchial lumen. Using this technique, no viral RNA was detected at 14 dpi in either group. 

In summary, our study examined the suitability of a small animal model to study SARS-CoV-2 infections and expands on determining the role that age might play in the differences with respect to disease progression. Intranasal infection of Syrian hamsters resulted in weight loss and robust virus replication in the upper and lower respiratory tract. A limitation of this study is the lack of an age-matched mock group for the older hamsters, which we are unable to include for legal reasons as we are bound under German law to follow the 3R principle. We further demonstrate that aged 32- to 34-week-old hamsters experienced higher and more consistent weight loss after intranasal infection, while body temperatures and virus replication in upper airways and lungs were similar between both age groups. Furthermore, we show that, using in situ hybridization, viral RNA was detectable in bronchial epithelial cells, type I and type II alveolar epithelial cells, and macrophages. All these cell types are potential targets of SARS-CoV-2 in human lung tissue; hence, the infection of hamsters of different ages seems to closely reflect what has been reported for human patients [28,39]. In contrast to SARS-CoV-2 titers, histopathological changes differed markedly between young and aged Syrian hamsters over time: younger animals launched more severe reactions at early time points after infection, while lesions and inflammation in the lungs became more pronounced and widespread at later time points in the elderly. Notably, age-related differences of SARS-Cov-2 infections have also been observed in non-human primates [17], and while this manuscript was under revision, in hamsters [40]. The study by Imai et al. also describes infections of Syrian hamsters of different age groups, and they also observed more robust body weight losses in older hamsters [40]. 

Based on the data presented here, we propose that comparative preclinical assessments of SARS-CoV-2 vaccines and other treatment options in young versus aged hamsters may yield valuable and relevant results, as this small animal model appears to mimic age-dependent differences in humans. The development of a humoral immune response emphasizes that hamsters are likely suitable for vaccination trials. Our observations also confirm that body weight loss is a readily quantifiable parameter that has proven very useful to measure disease severity in SARS-CoV-2 infection of Syrian hamsters. This makes the difference in body weight loss between age groups with more consistent losses in aged hamsters only more important as it provides an objective way to judge clinical efficacy of antiviral therapy or vaccination.

## Supplementary Materials

Supplementary materials can be found at https://www.mdpi.com/1999-4915/12/7/779/s1.
