# Supplementary material for: Age-Dependent Progression of SARS-CoV-2 Infection in Syrian Hamsters
Source: Viruses. 2020 Jul 20;12(7):779. doi: 10.3390/v12070779 (PMC7412213; doi:10.3390/v12070779)
Supplement: Supplementary file 1 [file viruses-12-00779-s001.pdf]

Supplementary Materials

Age-Dependent Progression of SARS-CoV-2 Infection in Syrian Hamsters

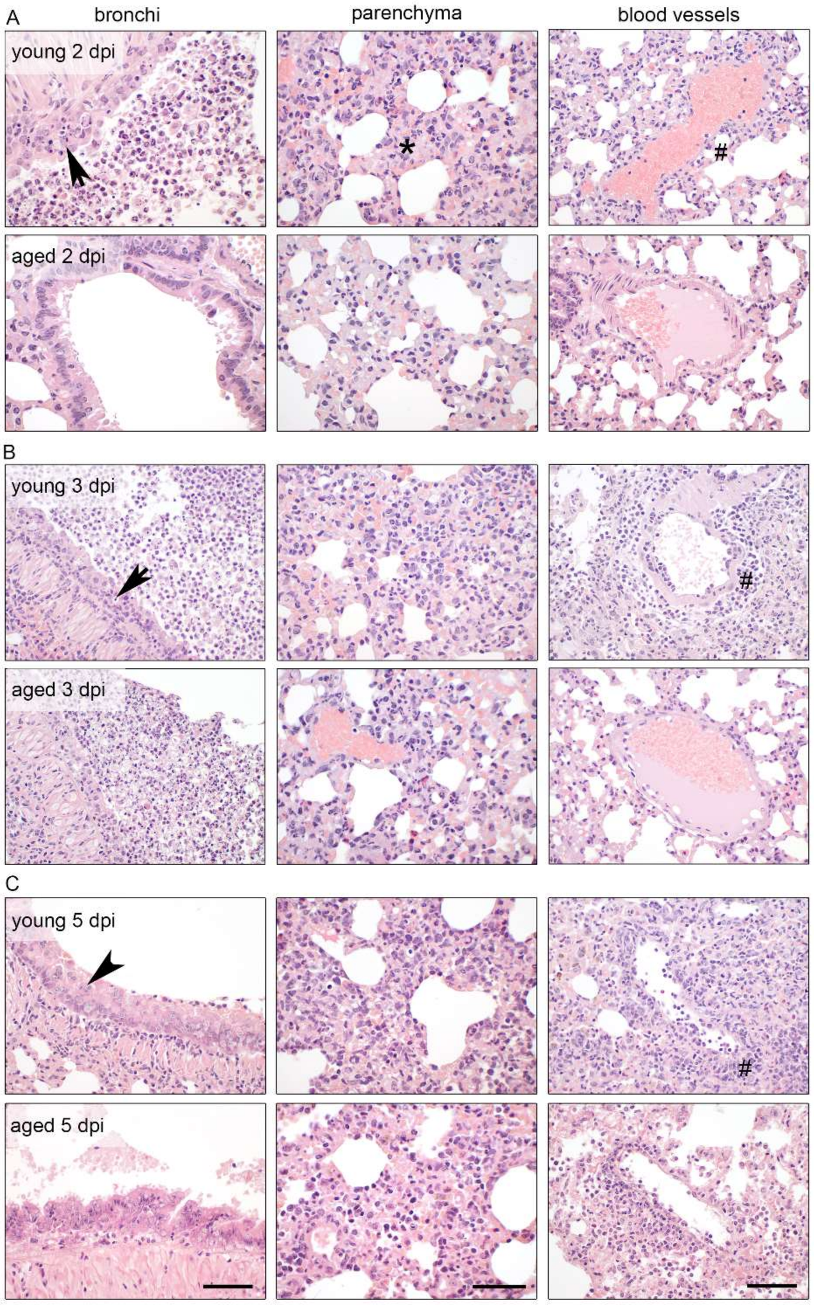

Figure S1: Histopathological comparison at early time points after SARS-COV-2 infection. (A) At 2 dpi, young animals developed marked necrotizing (left, arrow) and suppurative bronchitis (left) with cell debris and exudates filling the bronchial lumen, widely expanded interstitial tissue (center, asterisk) with infiltration of macrophages, neutrophils and lymphocytes and onset of perivascular lymphocytic cuffing (right, hash), which was milder or absent in aged hamsters. (B) At 3 dpi, necro-suppurative bronchitis (left, arrow) as well as interstitial pneumonia accelerated in both groups, while perivascular lymphocytic cuffing present only in young animals (right, hash). (C) At 5 dpi, hyperplasia of bronchial epithelia (left, arrowhead), interstitial pneumonia, and perivascular lymphocytic cuffing (right, hash) were identical in young and aged animals. Bars: left panels, 100 µm; center panels, 50 µm; right panels, 100 µm.

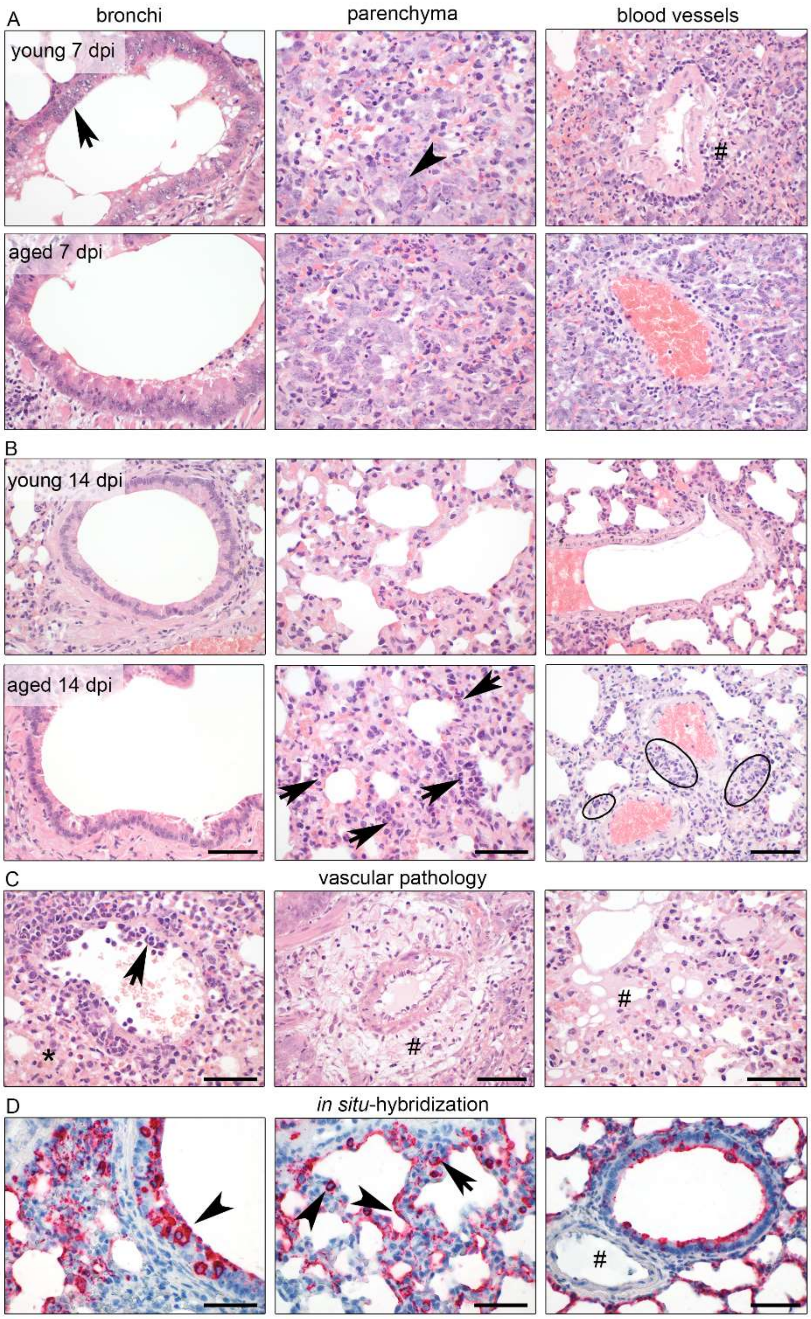

Figure S2: Histopathological comparison at late time points including vascular pathology and detection of SARS-COV-2 RNA. (A) At 7 dpi, dominant regeneration of bronchial epithelial cells (left, arrow) and type II alveolar epithelial cells (center, arrowhead) as well as prominent perivascular lymphocytic cuffing (right, hash) were present in both groups. (B) At 14 dpi, lungs of young animals showed only minimal mononuclear cell infiltration, restored tissue structures and largely resolved inflammation, while lungs of aged hamsters still had persistent tissue damage and active inflammation (center, arrow; right, oval). (C) Vascular pathology in both groups included endothelialitis (left, arrow), alveolar hemorrhage (left, asterisk), perivascular edema (center, hash) and alveolar edema (right, hash), shown here for representative animals of the aged group at 5 dpi. (D) Viral RNA was detected by *in situ* hybridization in bronchial epithelial cells (left, arrowhead), type I and II alveolar epithelial cells (center, arrowhead), and macrophages (center, arrow). Endothelial cells (right, hash) were not infected at the any of the time points in any of the lungs. Bars (A, B): left panels, 100 µm; center panels, 50 µm; right panels, 100 µm. Bars (C): left and right, 50 µm; center, 100 µm. Bars (D): left and center, 50 µm; right, 100 µm.

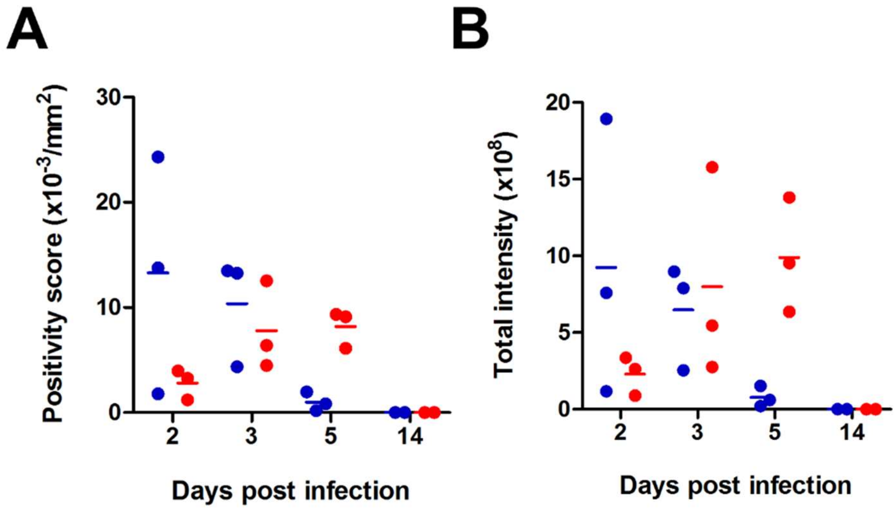

Figure S3: Digital quantitative comparison of *in situ*-hybridization signals for SARS-CoV-2 RNA in young (blue) and aged (red) hamsters. ISH signals were digitally quantified on whole slide scans of each animal using the Aperio positive pixel count algorithm. (A) Positivity score encompassing the number of positive pixels in relation to the total number of pixels per mm² tissue scan as well as (B) the total intensity of positive pixels were determined (n.s.; Mann–Whitney U test).

Table S1. Virus titers and RNA copies in lung homogenates (25 mg – RNA copies per 105 cellular transcripts), nasal washes, bucco-pharyngeal swabs, blood samples (25 µl), kidneys (25 mg), spleens (25 mg) and duodenums (25 mg), as well as serum neutralizing antibodies of each hamster.

#	group	sex	sample date	age	lung titers	RNA copies (lung)	RNA copies (wash)	RNA copies (swab)	RNA copies (blood)	RNA copies (kidney)	RNA copies (spleen)	RNA copies (duodenum)	Neutralizing antibody titer
**12**	uninf.	♀	2 dpi	young	0.00	0.00	0.00	n.d.	0.00	n.d.	n.d.	n.d.	n.d.
**19**	uninf.	♂	2 dpi	young	0.00	0.00	0.00	n.d.	0.00	n.d.	n.d.	n.d.	n.d.
**31**	uninf.	♀	2 dpi	young	0.00	0.00	0.00	n.d.	0.00	n.d.	n.d.	n.d.	n.d.
**10**	uninf.	♀	3 dpi	young	0.00	0.00	0.00	0.00	0.00	n.d.	n.d.	n.d.	n.d.
**13**	uninf.	♀	3 dpi	young	0.00	0.00	0.00	0.00	0.00	n.d.	n.d.	n.d.	n.d.
**27**	uninf.	♂	3 dpi	young	0.00	0.00	0.00	0.00	0.00	n.d.	n.d.	n.d.	n.d.
**3**	uninf.	♀	5 dpi	young	0.00	0.00	0.00	0.00	0.00	0.00	0.00	0.00	n.d.
**29**	uninf.	♂	5 dpi	young	0.00	0.00	0.00	0.00	0.00	0.00	0.00	0.00	n.d.
**33**	uninf.	♂	5 dpi	young	0.00	0.00	0.00	0.00	0.00	0.00	0.00	0.00	n.d.
**8**	uninf.	♀	14 dpi	young	0.00	0.00	0.00	0.00	0.00	n.d.	n.d.	n.d.	<1:8
**11**	uninf.	♀	14 dpi	young	0.00	0.00	0.00	0.00	0.00	n.d.	n.d.	n.d.	<1:8
**34**	uninf.	♀	14 dpi	young	0.00	0.00	0.00	0.00	0.00	n.d.	n.d.	n.d.	<1:8
**5**	inf.	♂	2 dpi	young	$3.00x10^{6}$	$3.46x10^{6}$	$3.45x10^{6}$	n.d.	$5.34x10^{5}$	$5.89x10^{3}$	0.00	$3.21x10^{3}$	n.d.
**15**	inf.	♂	2 dpi	young	$7.60x10^{6}$	$4.30x10^{6}$	$1.87x10^{5}$	n.d.	0.00	n.d.	n.d.	n.d.	n.d.
**35**	inf.	♂	2 dpi	young	$7.20x10^{6}$	$2.60x10^{7}$	$1.01x10^{2}$	n.d.	0.00	n.d.	n.d.	n.d.	n.d.
**2**	inf.	♀	3 dpi	young	$1.34x10^{6}$	$6.20x10^{5}$	$2.74x10^{4}$	$7.36x10^{4}$	$1.01x10^{2}$	n.d.	n.d.	n.d.	n.d.
**9**	inf.	♂	3 dpi	young	$1.03x10^{7}$	$3.14x10^{6}$	$2.17x10^{6}$	$4.26x10^{4}$	0.00	n.d.	n.d.	n.d.	n.d.
**26**	inf.	♀	3 dpi	young	$4.49x10^{5}$	$8.90x10^{5}$	$5.89x10^{4}$	$1.21x10^{5}$	0.00	n.d.	n.d.	n.d.	n.d.
**1**	inf.	♂	5 dpi	young	$4.33x10^{4}$	$1.29x10^{6}$	$6.80x10^{3}$	$1.88x10^{4}$	$7.56x10^{3}$	n.d.	n.d.	n.d.	n.d.
**24**	inf.	♀	5 dpi	young	$5.15x10^{3}$	$9.56x10^{5}$	$3.39x10^{3}$	$4.00x10^{5}$	0.00	0.00	0.00	0.00	n.d.
**36**	inf.	♀	5 dpi	young	$1.79x10^{4}$	$1.26x10^{6}$	$9.07x10^{6}$	$1.94x10^{4}$	0.00	0.00	0.00	0.00	n.d.

25	inf.	♂	7 dpi	young	0.00	9.18x10^{4}	9.72x10^{3}	4.73x10^{3}	6.43x10^{2}	n.d.	n.d.	n.d.	n.d.
14	inf.	♂	14 dpi	young	0.00	3.80x10^{2}	1.64x10^{2}	0.00	0.00	n.d.	n.d.	n.d.	1:512
30	inf.	♂	14 dpi	young	0.00	3.76x10^{2}	5.26x10^{1}	0.00	0.00	n.d.	n.d.	n.d.	1:203.2
18	inf.	♂	2 dpi	aged	7.80x10^{6}	2.20x10^{6}	6.24x10^{6}	n.d.	0.00	n.d.	n.d.	n.d.	n.d.
20	inf.	♂	2 dpi	aged	3.00x10^{6}	1.51x10^{7}	6.20x10^{6}	n.d.	0.00	n.d.	n.d.	n.d.	n.d.
32	inf.	♂	2 dpi	aged	3.80x10^{6}	9.52x10^{6}	8.84x10^{6}	n.d.	8.71x10^{2}	n.d.	n.d.	n.d.	n.d.
7	inf.	♀	3 dpi	aged	9.42x10^{4}	6.78x10^{6}	1.51x10^{5}	5.81x10^{4}	0.00	n.d.	n.d.	n.d.	n.d.
17	inf.	♂	3 dpi	aged	2.37x10^{6}	8.72x10^{5}	1.96x10^{7}	3.04x10^{5}	3.33x10^{5}	3.76x10^{3}	3.11x10^{3}	1.15x10^{4}	n.d.
21	inf.	♂	3 dpi	aged	1.80x10^{6}	3.38x10^{5}	2.39x10^{6}	1.46x10^{7}	0.00	n.d.	n.d.	n.d.	n.d.
6	inf.	♀	5 dpi	aged	2.00x10^{4}	1.83x10^{6}	1.35x10^{4}	1.51x10^{4}	0.00	n.d.	n.d.	n.d.	n.d.
23	inf.	♀	5 dpi	aged	3.45x10^{4}	2.84x10^{6}	4.30x10^{4}	2.86x10^{4}	0.00	0.00	9.27x10^{1}	6.95x10^{2}	n.d.
28	inf.	♂	5 dpi	aged	4.25x10^{4}	2.44x10^{6}	9.55x10^{5}	5.09x10^{4}	0.00	2.56x10^{2}	0.00	2.93x10^{2}	n.d.
16	inf.	♀	7 dpi	aged	0.00	2.58x10^{3}	6.19x10^{3}	7.45x10^{5}	0.00	n.d.	n.d.	n.d.	n.d.
4	inf.	♀	14 dpi	aged	0.00	4.08x10^{2}	0.00	0.00	0.00	n.d.	n.d.	n.d.	1:128
22	inf.	♀	14 dpi	aged	0.00	8.98x10^{2}	0.00	0.00	0.00	n.d.	n.d.	n.d.	1:80.6

Table S2. Oligonucleotides used in this study.

**Primer/probe**	**Sequence 5′–3′**
SARS-CoV-2 forward	ACAGGTACGTTAATAGTTAATAGCGT
SARS-CoV-2 reverse	ATATTGCAGCAGTACGCACACA
SARS-CoV-2 probe	FAM-ACACTAGCCATCCTTACTGCGCTTCG-BHQ
RPL-18 forward	GTTTATGAGTCGCACTAACCG
RPL-18 reverse	TGTTCTCTCGGCCAGGAA
RPL-18 probe	FAM-TCTGTCCCTGTCCCGGATGATC-BHQ
